# Supplementary material for: Probing the ionic defect landscape in halide perovskite solar cells
Source: Nat Commun. 2020 Nov 30;11:6098. doi: 10.1038/s41467-020-19769-8 (PMC7705665; doi:10.1038/s41467-020-19769-8)
Supplement: Supplementary file 1 — Supplementary Information [file 41467_2020_19769_MOESM1_ESM.pdf]

Supplementary Information: Probing the ionic defect landscape in halide perovskite solar cells

Sebastian Reichert,[1] Qingzhi An,[2,3] Young-Won Woo,[4,5] Aron Walsh,[4,5] Yana Vaynzof,[2,3] and Carsten Deibel[1,a]

[1] *Institut für Physik, Technische Universität Chemnitz, 09126 Chemnitz, Germany*
[2] *Kirchhoff-Institut für Physik and Centre for Advanced Materials, Ruprecht-Karls-Universität Heidelberg, Im Neuenheimer Feld 227, 69120 Heidelberg, Germany*
[3] *Integrated Centre for Applied Physics and Photonic Materials and Centre for Advancing Electronics Dresden (cfaed), Technical University of Dresden, Nöthnitzer Straße 61, 01187 Dresden, Germany*
[4] *Department of Materials, Imperial College London, Exhibition Road, London SW7 2AZ, UK*
[5] *Department of Materials Science and Engineering, Yonsei University, Seoul 03722, Korea*

(Dated: October 27, 2020)

[a] Corresponding author: Carsten Deibel (deibel@physik.tu-chemnitz.de)

SUPPLEMENTARY NOTE 1. CURRENT DENSITY-VOLTAGE (JV) CHARACTERISTICS

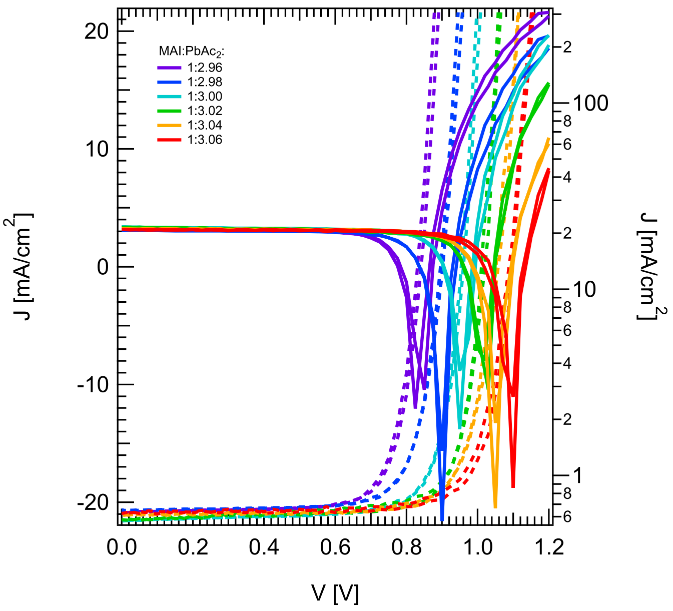

Supplementary Fig. 1. Current density–voltage (jV) measurement of the $MAPbI_3$ solar cells with stoichiometric variation at 1 sun in linear (left axis) and logarithm representation (right axis).

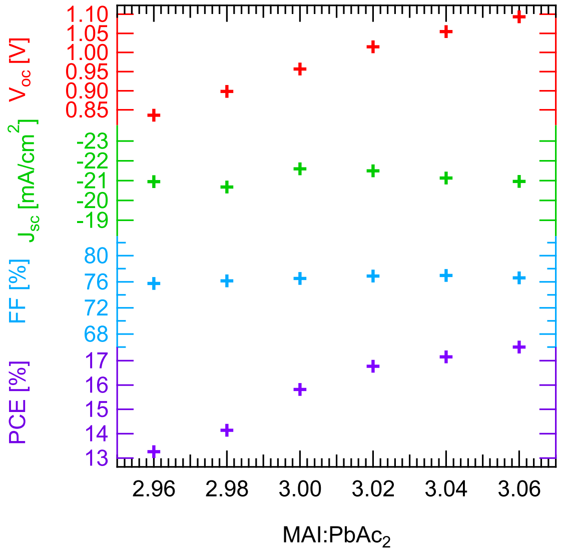

Supplementary Fig. 2. Current density characteristics are plotted over the stoichiometric ratio MAI:$PbAc_2$: Open circuit voltage V_{oc} (red), fill factor FF (blue) as well as power conversion efficiency PCE (violet) increase with higher stoichiometric ratio whereas short circuit current J_{sc} (green) keeps constant.

SUPPLEMENTARY NOTE 2. CAPACITANCE-VOLTAGE MEASUREMENT

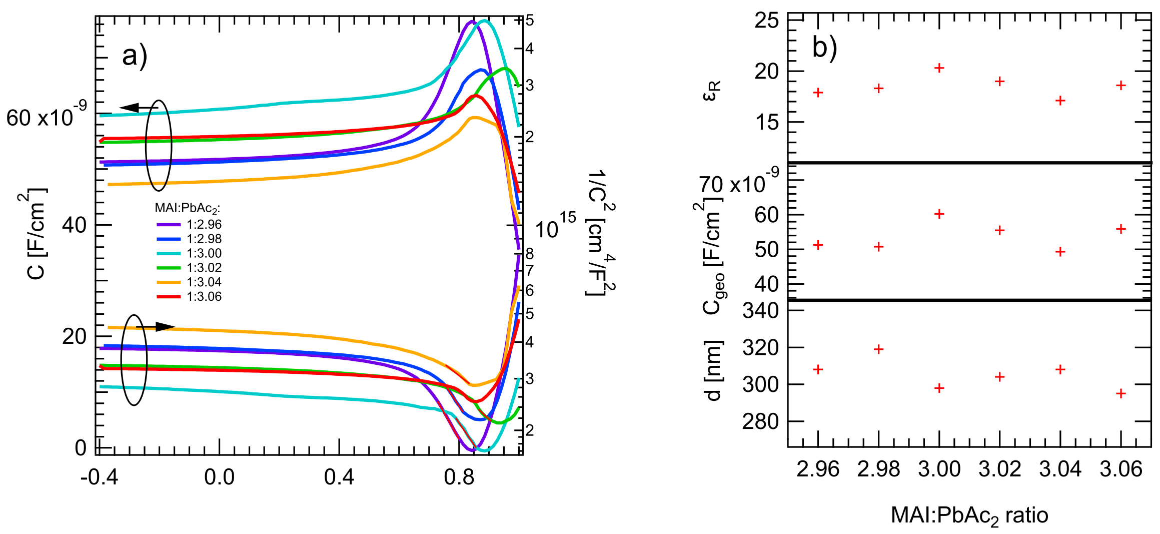

Supplementary Fig. 3. a) Capacitance–voltage measurement on the perovskite solar cells with variation of precursor stoichiometry at room temperature with an ac frequency of 80 kHz. The data were evaluated according to the Mott–Schottky approach by plotting $1/C^2$ and fitting the linear part at around 0.8 V which correspond to the depletion capacitance. b) Relative Permittivity can be calculated from geometrical capacitance C_{geo} obtained from negative voltage part of a) and film thickness d by AFM measurements using Eqn. (2).

SUPPLEMENTARY NOTE 3. IS MEASUREMENTS

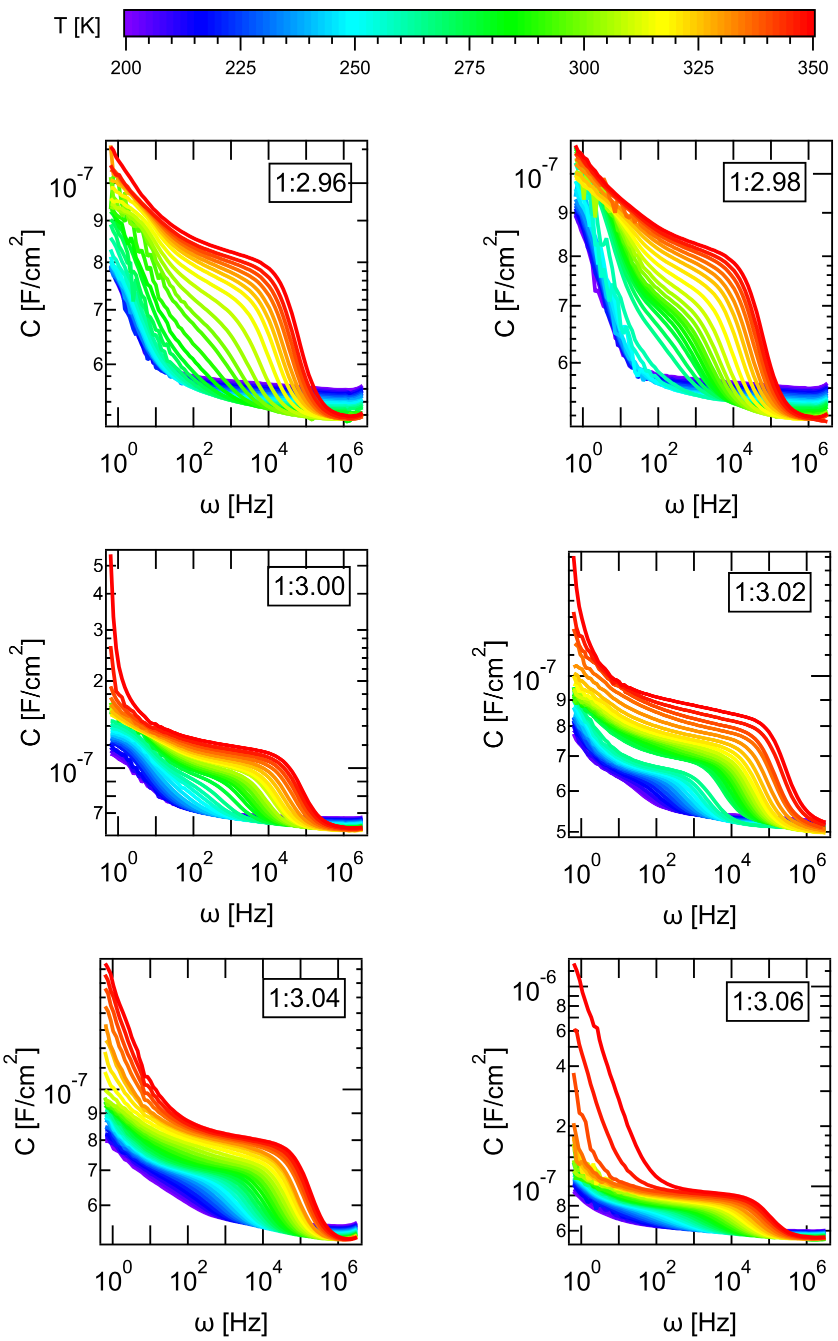

Supplementary Fig. 4. Impedance spectroscopy measurements of the perovskite solar cells with different precursor stoichiometry for temperature variations between 200 K and 350 K in 5 K steps. From a) to f) changes the stoichiometry from 2.96 to 3.06 in 0.02 steps.

SUPPLEMENTARY NOTE 4. IS EVALUATION

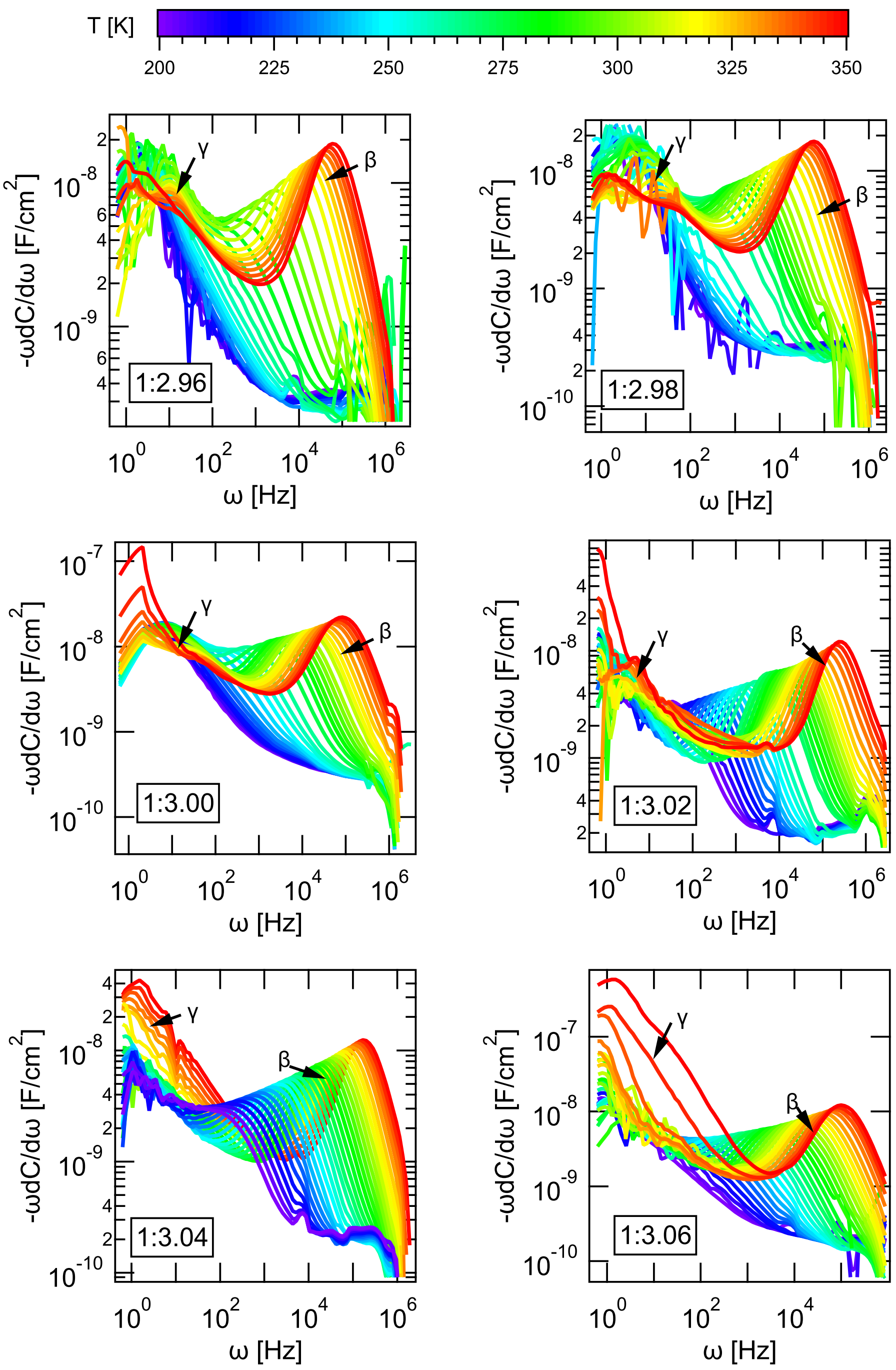

Supplementary Fig. 5. Evaluation of IS measurements of Supplementary Fig. 4 by calculating the derivation $-\omega dC/d\omega$. The maximum of each peak correspond to the migration rates which were plotted in Supplementary Fig. 6.

SUPPLEMENTARY NOTE 5. ARRHENIUS PLOT OF IS AND DLTS MEASUREMENTS

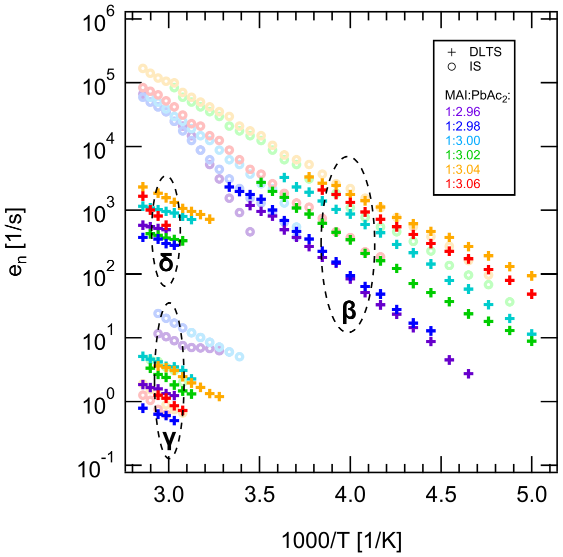

Supplementary Fig. 6. Arrhenius plot of migration rates extracted from IS and DLTS measurements for all six perovskite solar cells with different precursor stoichiometry. Migration rates were attributed to belong to three different ionic defects, labelled with β (crosses), γ (circles) and δ (stars). Defect δ was only accessible by DLTS measurements, whereas migration rates of β and γ measured by IS and DLTS show good agreement.

SUPPLEMENTARY NOTE 6. DLTS MEASUREMENTS

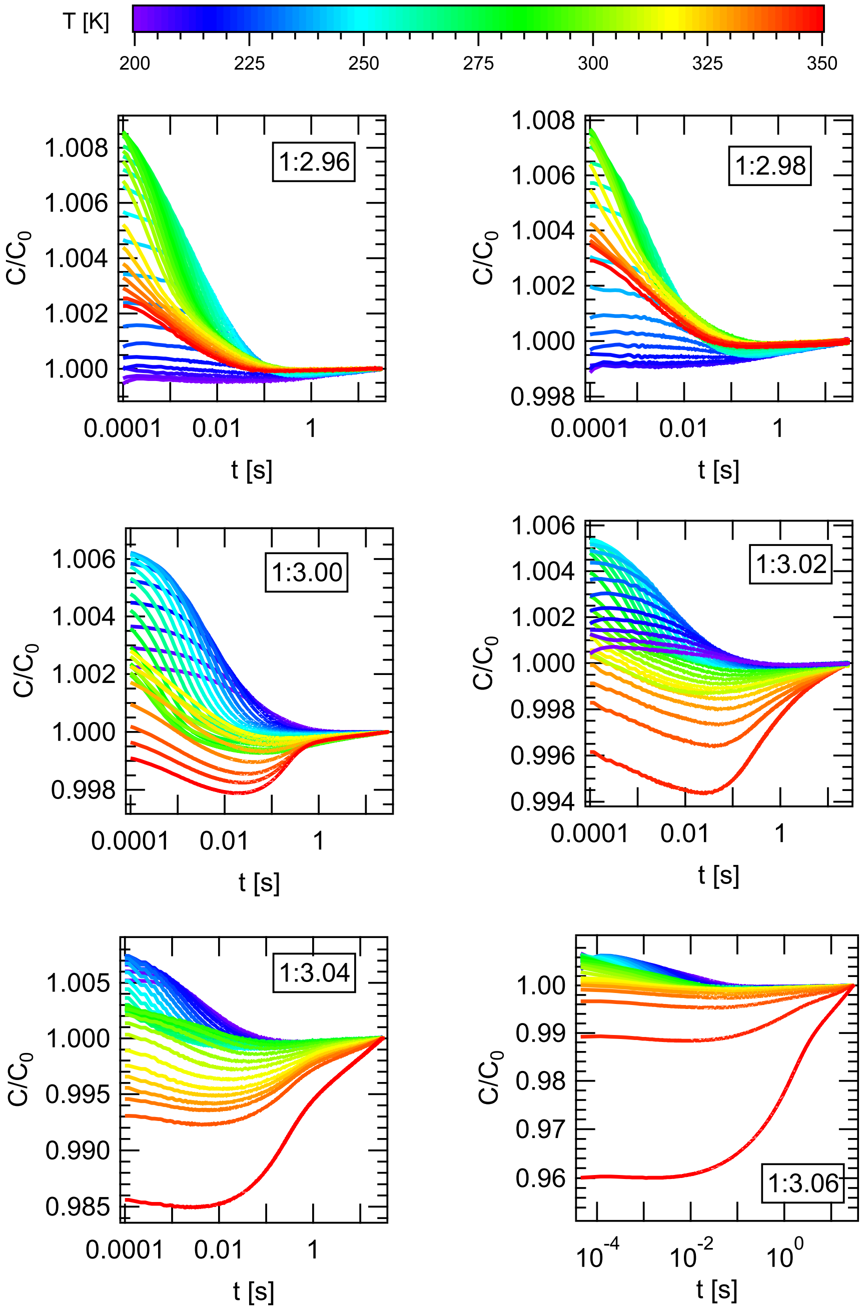

Supplementary Fig. 7. DLTS measurements for different temperatures from 200 K to 350 K in 5K steps for all perovskite solar cells with different stoichiometric ratio. The transients were normalized with the equilibrium capacitance C_0 and measured over 30 s.

SUPPLEMENTARY NOTE 7. BOXCAR EVALUATION OF THE DLTS MEASUREMENTS

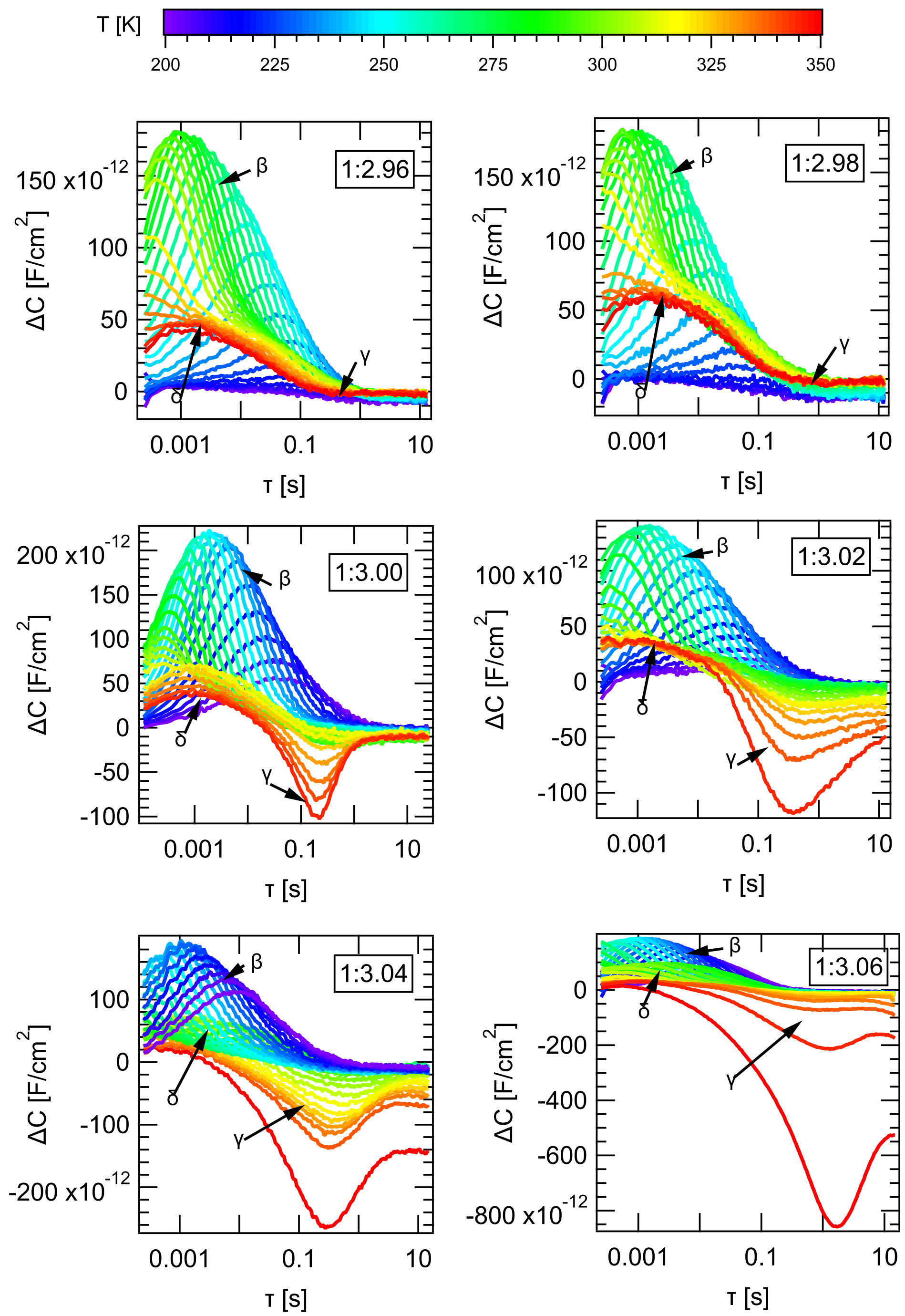

Supplementary Fig. 8. Boxcar evaluation of the DLTS measurements (Supplementary Fig. 7) with rate window $t_2/t_1 = 5$. Visible are three different defects β, γ and δ.

SUPPLEMENTARY NOTE 8. RELATION BETWEEN E_A, D_{300K} AND N_{ion}

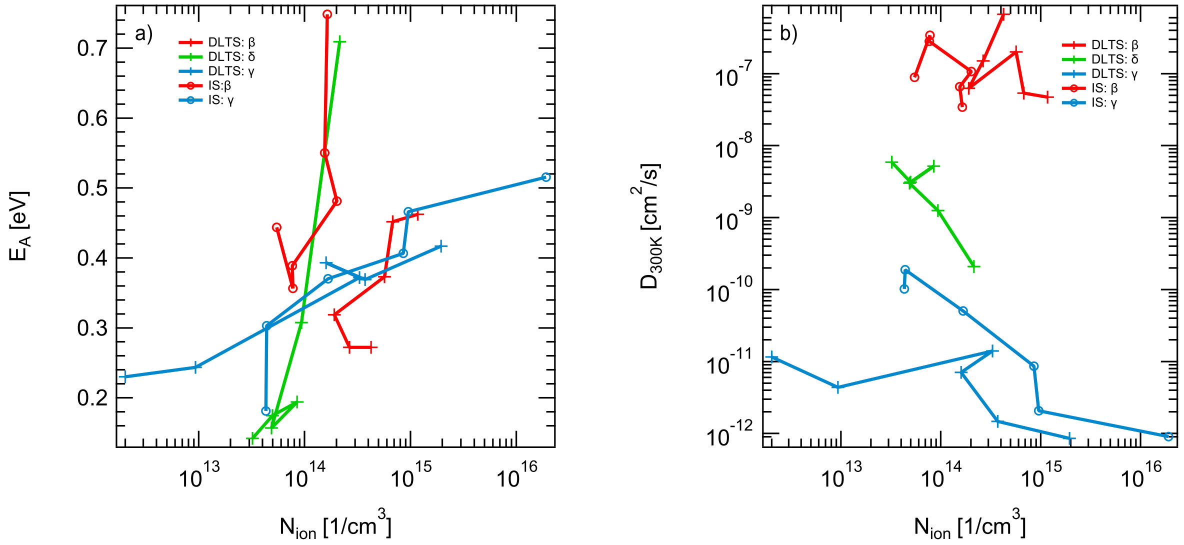

Supplementary Fig. 9. Plot of the activation energy E_A (a) and diffusion coefficient at 300 K D_{300K} (b) over the ionic defect concentration N_{ion} extracted from DLTS (crosses) and IS (circles). For all ionic species β (red), γ (blue) and δ (green), E_A seems to increase with the ionic defect concentration whereas D_{300K} decreases.

SUPPLEMENTARY NOTE 9. LITERATURE COMPARISON OF REPORTED DEFECT PARAMETERS

Supplementary Tab. 1. Literature comparison of reported defect parameters including activation energy E_A, ion concentration N_{ion} and charge type.

reference	perovskite	method	defect name	charge type	E_A (eV)	N_{ion} (cm^{-3})
Samiee et al.[1]	$CH_3NH_3PbI_xCl_{1-x}$	IS	A	—	0.240	$3 \cdot 10^{16}$
			B	—	0.660	$3 \cdot 10^{16}$
Yang et al.[2]	$FAPbI_3$	DLTS	A1	majoritiy/cation	0.820	—
			A2	majoritiy/cation	0.780	$5 \cdot 10^{14}$
			A3	majoritiy/cation	0.460	—
Rosenberg et al.[3]	$MAPbBr_3$ (single crystals)	current DLTS	E1	—	0.204	10^9
			E2	—	0.167	10^8
Xu et al.[4]	$FAPbI_3$	IS	control	—	0.400	$6 \cdot 10^{14}$
			passivated	—	0.140	$5 \cdot 10^{14}$
Futscher et al.[5]	$MAPbI_3$	DLTS	A1	anion	0.290	$1 \cdot 10^{15}$
			C1	cation	0.900 (0.460)	$1 \cdot 10^{16}$
			C2	cation	0.390	$5 \cdot 10^{15}$

SUPPLEMENTARY NOTE 10. UNDERSTANDING THE IMPACT OF SMALL ION DENSITIES ON THE ELECTRONIC LANDSCAPE

As reported in several studies,[6, 7] ion densities in the range of the charge carrier density have an impact on the electronic landscape by causing interfacial bend banding and screening partly the internal electric field. To calculate the voltage drop caused by ion accumulation (i.e. the ion density N_{ion}), it is very important whether it can be assumed that only one species, e.g. cations or both species (anions and cations) are considered mobile. Accordingly, one can differentiate between a one-ion model and a two-ion model respectively. Eqn. (9) represents the potential drop in the two-ion model whereas for the one-ion model an approach without series connection of the double layer capacitance can be found[6]:

$$\Delta V_{\text{bi}} = \frac{\sqrt{N_{\text{ion}} \varepsilon_{\text{R}} \varepsilon_0 k_{\text{B}} T}}{\Delta C} \tag{S1}$$

where ΔC corresponds to the capacitance step of the IS spectra and ΔV_{bi} refers to the potential drop by ion accumulation in accordance with our calculation of Eqn. (8). We have demonstrated this calculation for the sample with the largest potential drop with stoichiometry of 3.06 according to our calculation with Eqn. (9) and Fig. (1) and the approach, that only cations are moveable in the one-ion model:

Supplementary Tab. 2. a) Calculation of the potential drop by using the two-ion model according to Eqn. (9). b) Calculation of the ion density using the one-ion model and the same potential drop as in a). c) Comparison with Almora et al.[6].

	a) two-ion model (Eqn. (9))	b) one-ion model (Eqn. (S1))	c) One-ion model with estimation by Almora et al.[6]
N_{ion_C}	$1.9 \cdot 10^{16}$ cm−3	$2.7 \cdot 10^{18}$ cm^{-3}	$2.4 \cdot 10^{17}$ cm^{-3}
N_{ion_A}	$1.3 \cdot 10^{14}$ cm^{-3}		
ΔC_{C}	$4.1 \cdot 10^{-7}$ F/cm^2	$4.1 \cdot 10^{-7}$ F/cm^2	$2 \cdot 10^{-6}$ F/cm^2
ΔC_{A}	$3.4 \cdot 10^{-8}$ F/cm^2		
ε_{R}	19	19	32.5
ΔV_{bi}	326 mV	326 mV	27 mV

All calculations were carried out for room temperature. We calculated the necessary ion density for the one-ion model in column b) to cause the same potential drop ΔV_{bi} as determined in the two-ion model (Fig. 1) by keeping the experimental observable ΔC and ε_{R} constant. As shown in column a) and b), the ion densities can be significantly different to impact the electronic landscape expressed by the same voltage drop, depending on which model was applied for the respective perovskite. The difference originates from the fact that in the case of the one-ion model no double layer capacitance is assumed, i.e. no serial connection of the capacitive contributions of different ionic species. In column c) we compared our calculation with the estimation of the voltage drop according to Almora et al.[6]. It shows the influence of space charge capacitance in relation to ion density on the calculation of the potential drop and confirms our findings.

SUPPLEMENTARY REFERENCES

[1] M. Samiee, S. Konduri, B. Ganapathy, R. Kottokkaran, H. A. Abbas, A. Kitahara, P. Joshi, L. Zhang, M. Noack, and V. Dalal, Defect density and dielectric constant in perovskite solar cells, Applied Physics Letters **105**, 153502 (2014).

[2] W. S. Yang, B.-W. Park, E. H. Jung, N. J. Jeon, Y. C. Kim, D. U. Lee, S. S. Shin, J. Seo, E. K. Kim, J. H. Noh, and S. I. Seok, Iodide management in formamidinium-lead-halide–based perovskite layers for efficient solar cells, Science **356**, 1376 (2017).

[3] J. W. Rosenberg, M. J. Legodi, Y. Rakita, D. Cahen, and M. Diale, Laplace current deep level transient spectroscopy measurements of defect states in methylammonium lead bromide single crystals, Journal of Applied Physics **122**, 145701 (2017).

[4] W. Xu, Q. Hu, S. Bai, C. Bao, Y. Miao, Z. Yuan, T. Borzda, A. J. Barker, E. Tyukalova, Z. Hu, M. Kawecki, H. Wang, Z. Yan, X. Liu, X. Shi, K. Uvdal, M. Fahlman, W. Zhang, M. Duchamp, J.-M. Liu, A. Petrozza, J. Wang, L.-M. Liu, W. Huang, and F. Gao, Rational molecular passivation for high-performance perovskite light-emitting diodes, Nature Photonics **13**, 418 (2019).

[5] M. H. Futscher, J. M. Lee, L. McGovern, L. A. Muscarella, T. Wang, M. I. Haider, A. Fakharuddin, L. Schmidt-Mende, and B. Ehrler, Quantification of ion migration in $CH_3NH_3PbI_3$ perovskite solar cells by transient capacitance measurements, Materials Horizons **6**, 1497 (2019).

[6] O. Almora, I. Zarazua, E. Mas-Marza, I. Mora-Sero, J. Bisquert, and G. Garcia-Belmonte, Capacitive dark currents, hysteresis, and electrode polarization in lead halide perovskite solar cells, The Journal of Physical Chemistry Letters **6**, 1645 (2015).

[7] L. Bertoluzzi, C. C. Boyd, N. Rolston, J. Xu, R. Prasanna, B. C. O'Regan, and M. D. McGehee, Mobile ion concentration measurement and open-access band diagram simulation platform for halide perovskite solar cells, Joule (2020).
